# Supplementary material for: E-cadherin can limit the transforming properties of activating β-catenin mutations
Source: EMBO J. 2015 Aug 3;34(18):2321–33. doi: 10.15252/embj.201591739 (PMC4570519; doi:10.15252/embj.201591739)

E-cadherin can limit the transforming properties of activating β–catenin mutations

Appendix

1. Appendix figure legends
2. Appendix supplementary methods
3. Appendix figures S1-S6

Fig. S1 Mutation of β-catenin in stem cells leads to tumour formation.
Survival curve of *Lgr5Cre*^{ER} aged cohorts. Mice were sampled when signs of intestinal tumour burden where apparent. Mutation of a single β-catenin mutation in *Lgr5* positive stem cells (*Lgr5Cre*^{ER} *Catnb*$^{lox(ex3)/+}$) led to tumour formation. Activation of 2 copies of β-catenin (*Lgr5Cre*^{ER} *Catnb*$^{lox(ex3)/lox(ex3)}$) led to a significant (log-rank test p=0.004) decrease in tumour-free survival.

Fig. S2 Haploinsufficiency for E-cadherin reduces E-cadherin expression and the number of E-cadherin:β-catenin complexes.
QRT-PCR for E-cadherin and β-catenin from small intestinal tissue of wildtype and *VilCre*^{ER} *Cdh1*$^{fl/+}$ mice shows a reduction only in E-cadherin expression (Cdh1) but not β-catenin (Ctnnb1), method=ΔΔCt, N=3 mice (B) Proximity ligation assay for E-cadherin and β-catenin shows a reduction in the number of complexes in both the small intestine and the colon of *VilCre*^{ER} *Cdh1*$^{fl/+}$ compared to wildtype mice (WT). For each mouse (N=3), at least 10 crypts per tissue were analysed to calculate the mean, statistics: one-sided Mann-Whitney U.

Fig. S3 Comparison of Wnt target gene expression after APC loss and mutation of β-catenin and reduction of E-cadherin levels
QRT-PCR of small intestine tissue for Wnt target *genes* in wildtype *VilCre*^{ER} *Catnb*$^{+/+}$ (WT), *VilCre*^{ER} *Catnb* $^{lox(ex3)/+}$ *(Catnb), VilCre*^{ER} *Catnb* $^{lox(ex3)/+}$ *Cdh1*$^{fl/+}$ (Catnb Cdh1) and *VilCre*^{ER} *Apc*$^{fl/fl}$ (Apc) sampled at timepoint indicated. *VilCre*^{ER} *Catnb* $^{lox(ex3)/+}$>25 days after induction were sampled due to signs of sickness (day 25, day 25 and day 29). Expression of mRNA ($2^{(-dCt)}$) calculated relative to GAPDH. N=3 mice per genotype, per timepoint (apart WT *Axin2*, *Lgr5* N=2).

Fig. S4 Mutations in *APC* and β-catenin in human colorectal cancer (CRC).
Analysis of *APC* and *CTNNB1* mutations in cbioportal.org. (A, B) Mutations in Human Colorectal Cancer in *APC* and *CTNNB1*. (C) Mutations in *CTNNB1* in Human Liver Hepatocellular Carcinoma (Ahn et al, 2014). Red circle highlights the hotspot of activating mutations in exon 3. Red dot represents nonsense mutations, green dot missense mutations. (D) Table with frequency of Wnt pathway activation in different cancers.

Fig. S5 Efficancy of small intestinal and colonic crypts *in vitro*.
(A) *In vitro* growth of crypts small intestine and colon from mice as indicated at day 5 post induction. Quantification of sphere forming efficiency of *AhCreER Catnb*$^{lox(ex3)/+}$ *AhCreER Catnb*$^{lox(ex3)/+}$ *Cdh1*$^{fl/+}$ without R-spo1, day 5 post induction. N=3 mice per genotype. The data for the small intestine is the same as in Fig. 5. (B) Crypts from wildtype mice were purified with 2 mM (small intestine) and 25 mM EDTA (colon), embedded and analysed for E-cadherin:β-catenin complexes using PLA. N=3 mice, mean of 10 crypts per mouse were used.

Fig. S6 PLA of *VilCre^ER^ Apc^fl/fl^* crypts from small intestine and colon *in vitro* and *in vivo* .
(A) The small intestine and the colon of *VilCre*^{ER} *Apc*$^{fl/fl}$, sampled at day 4 after induction. Mice were analysed for E-cadherin:β-catenin complexes by PLA (B) The spheres from the small intestine and colon of *VilCre*^{ER} *Apc*$^{fl/fl}$ mice (passage 2-3) were embedded and analysed by PLA for E-cadherin:β-catenin complexes. N=3 mice, mean of 10 crypts per mouse sample were used (A) and (B).

Supplementary Methods

Cre induction regimes

Recombination by *AhCre*^{ER} was induced with two intraperitoneal (IP) injections of 2 mg β-naphthoflavone/tamoxifen per day on two consecutive days. Recombination by *VilCre*^{ER} transgene was induced with one injection (IP) of 2 mg tamoxifen on two consecutive days. Recombination by expression of the *AhCre* transgene was induced by three injections (IP) of 2 mg β-naphthoflavone in a single day spaced 4 hours apart. *Lgr5Cre*^{ER} transgene expression was induced by a single administration of 2 mg tamoxifen (IP). Cohort mice were aged until they showed signs of intestinal tumourigenesis, indicated by anaemia, and/or weight loss.

QRT-PCR from mouse samples

500 ng of total RNA isolated from small intestinal tissue (Qiagen RNeasy) was reverse transcribed to cDNA and qPCR was carried out according to manufacturer's protocols (Finnzyme). The expression was normalised to *Gapdh*. Following primers were used:

**Gapdh F**	**GAAGGCCGGGGCCCACTTGA**
**Gapdh R**	**CTGGGTGGCAGTGATGGCATGG**
**Axin2 F**	**GCGACGCACTGACCGACGAT**

**Axin2 R**	**GCAGGCGGTGGGTTCTCGGA**
**Lgr5 F**	**GAC AAT GCT CTC ACA GAC**
**Lgr5 R**	**GGA GTG GAT TCT ATT ATT ATG G**
**Bcat F**	**ATCTTAAGCCCTCGCTCGGT**
**Bcat R**	**CTTCAGGTACCCTCAGGCCC**
**Ecad**	**ACTGTGAAGGGACGGTCAAC**
**Ecad**	**GGAGCAGCAGGATCAGAATC**
**cd44 F**	**CAC ATA TTG CTT CAA TGC CTC AG**
**cd44 R**	**CCA TCA CGG TTG ACA ATA GTT ATG**
**cMyc F**	**CCC AAA TCC TGT ACC TCG TC**
**cMyc R**	**TTG CCT CTT CTC CAC AGA CA**

Appendix Fig. S1

A

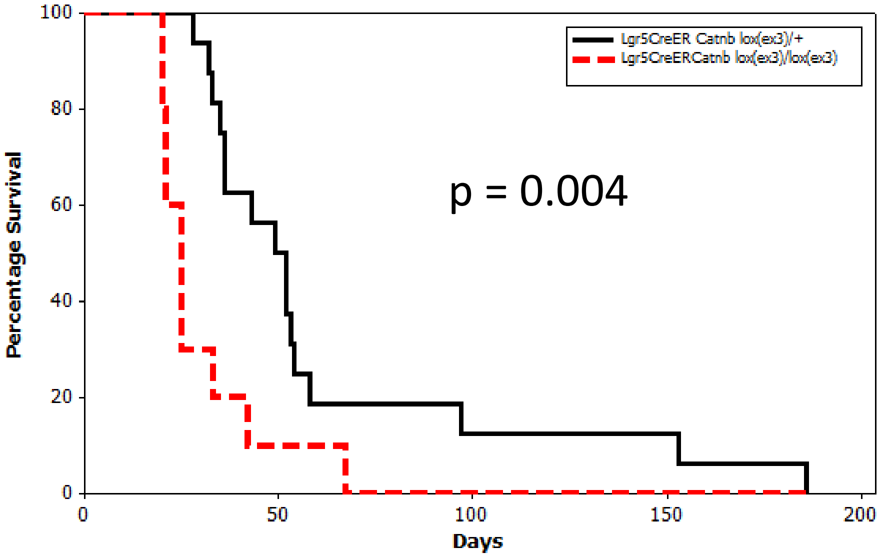

Appendix Fig. S2

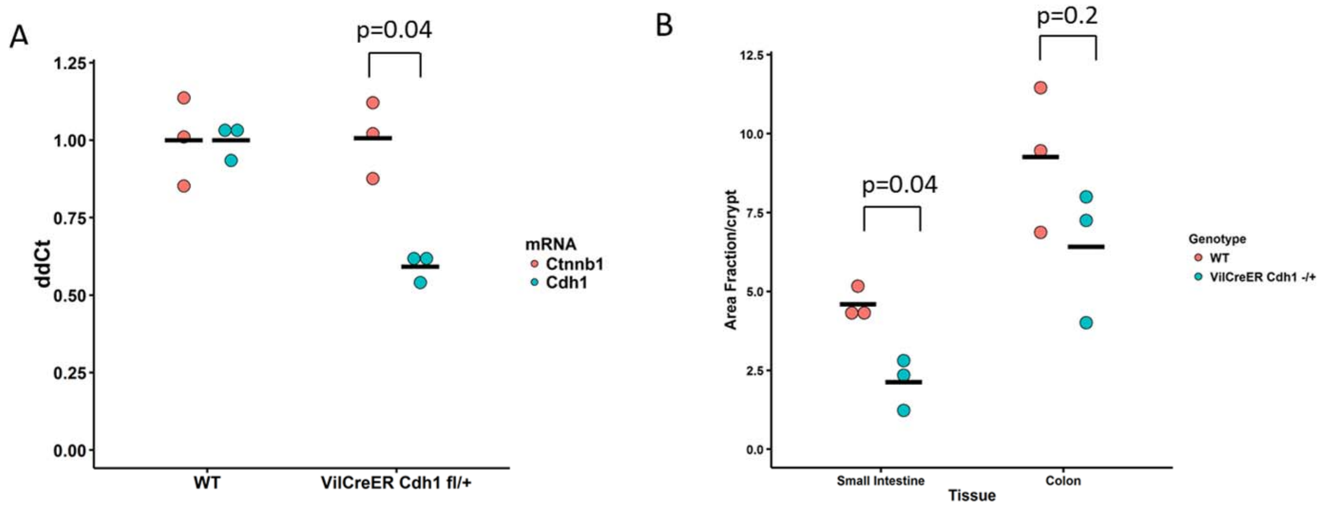

Appendix Fig. S3

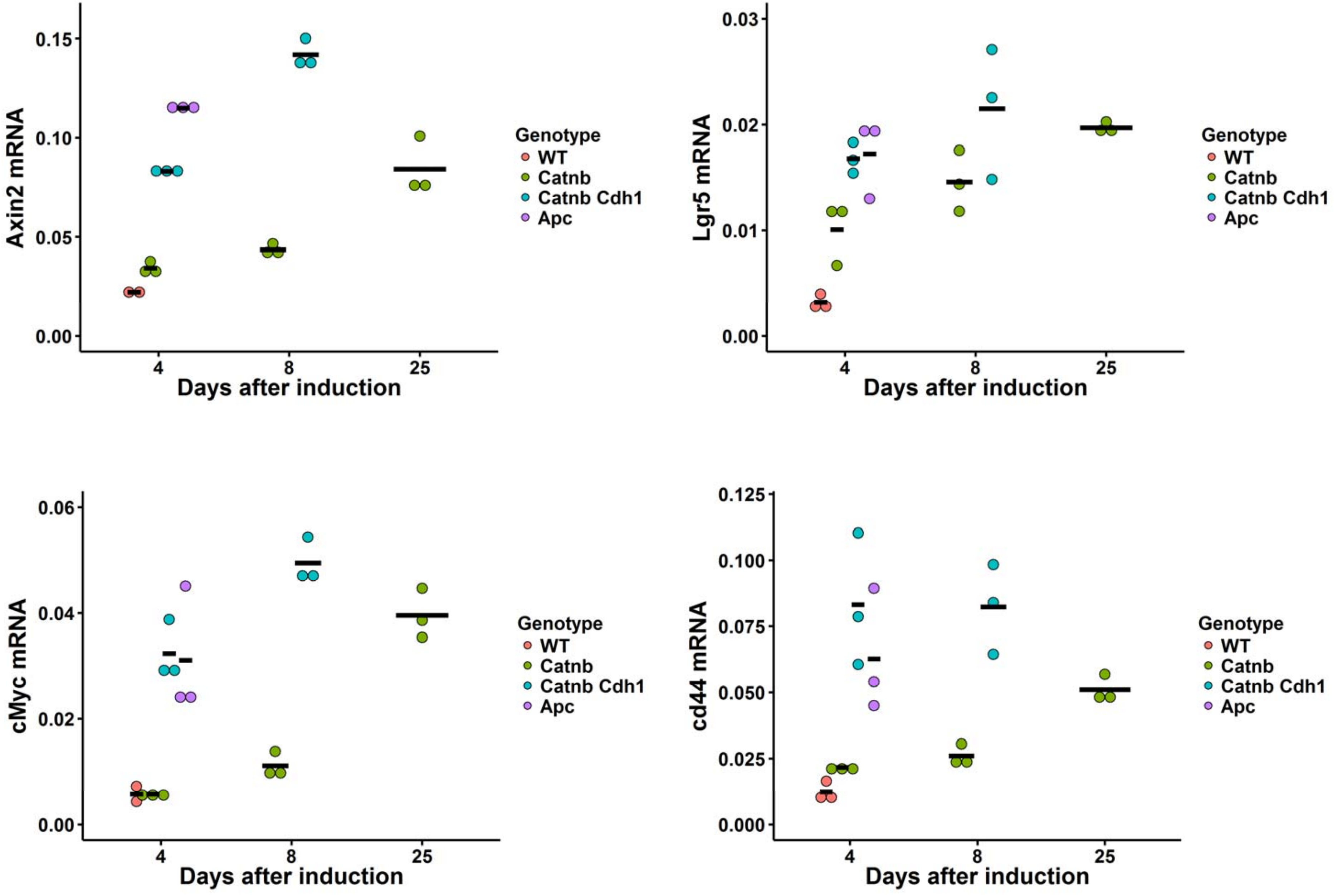

Appendix Fig. S4

A

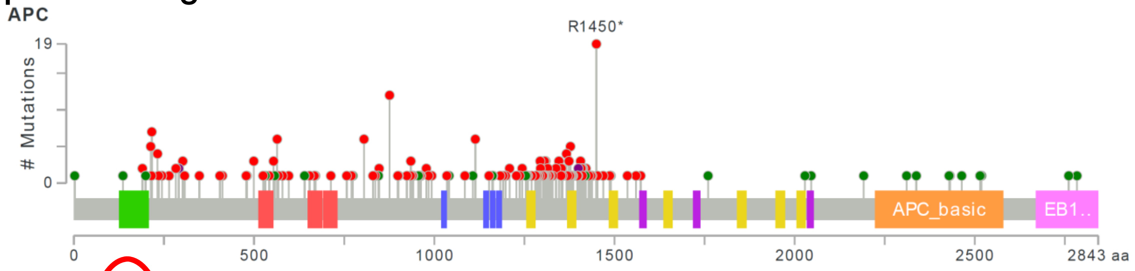

B

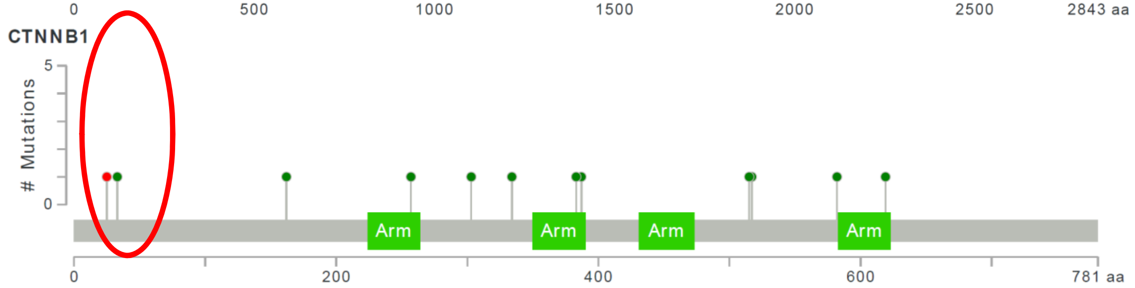

C

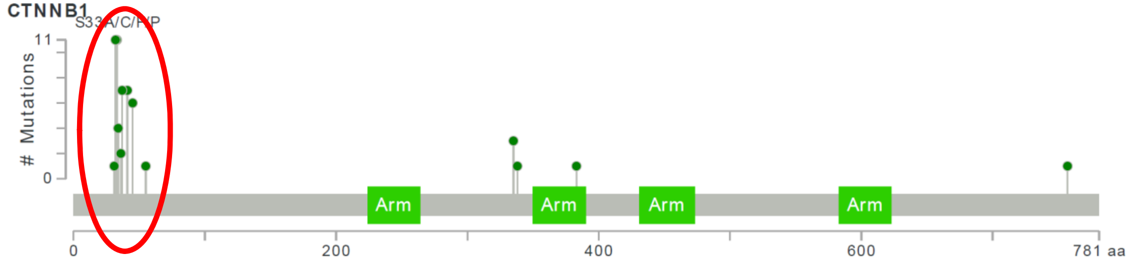

D

Tissue	Gene	Mutated Samples
Colorectal Adenocarcinoma (TCGA)	APC	168/224 (75%)
	CTNNB1	11/224 (4.9%) – **within exon 3: 2/224 (0.9%)**
	GSK3A	2/224 (<1%)
	GKS3B	6/224 (3%)
Liver Hepatocellular Carcinoma (AMC)	APC	4/231 (2%)
	CTNNB1	52/231 (22.5%) – **Within exon 3: 46/231 (19.9%)**
Pancreas – Solid pseudopapillary carcinoma (COSMIC)	APC	0/122 (0%)
	CTNNB1	110/122 (90.16 %) – **Within exon 3: 110/122 (90.16%)**

Appendix Fig. S5

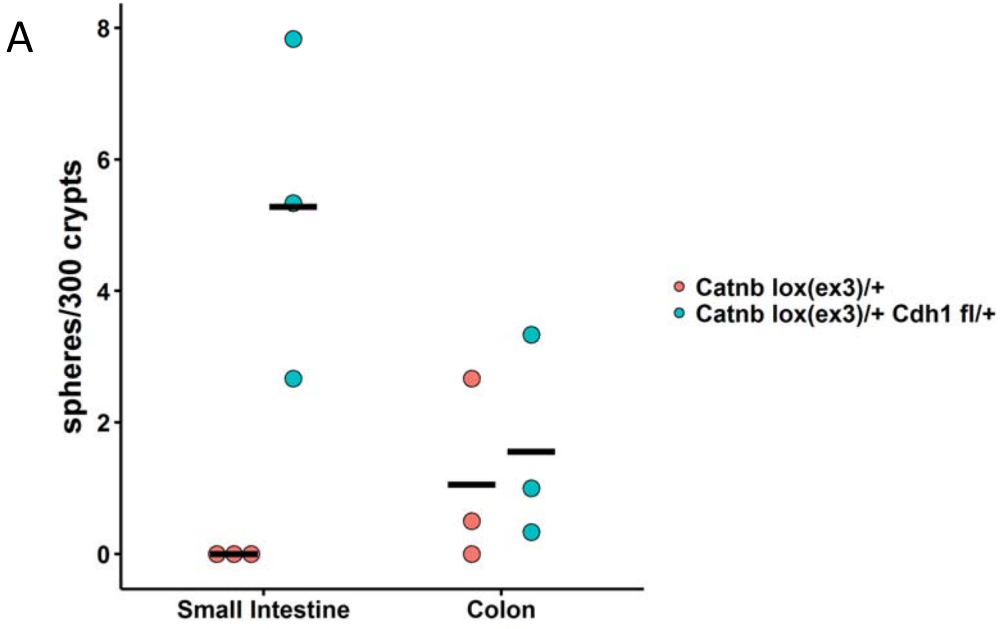

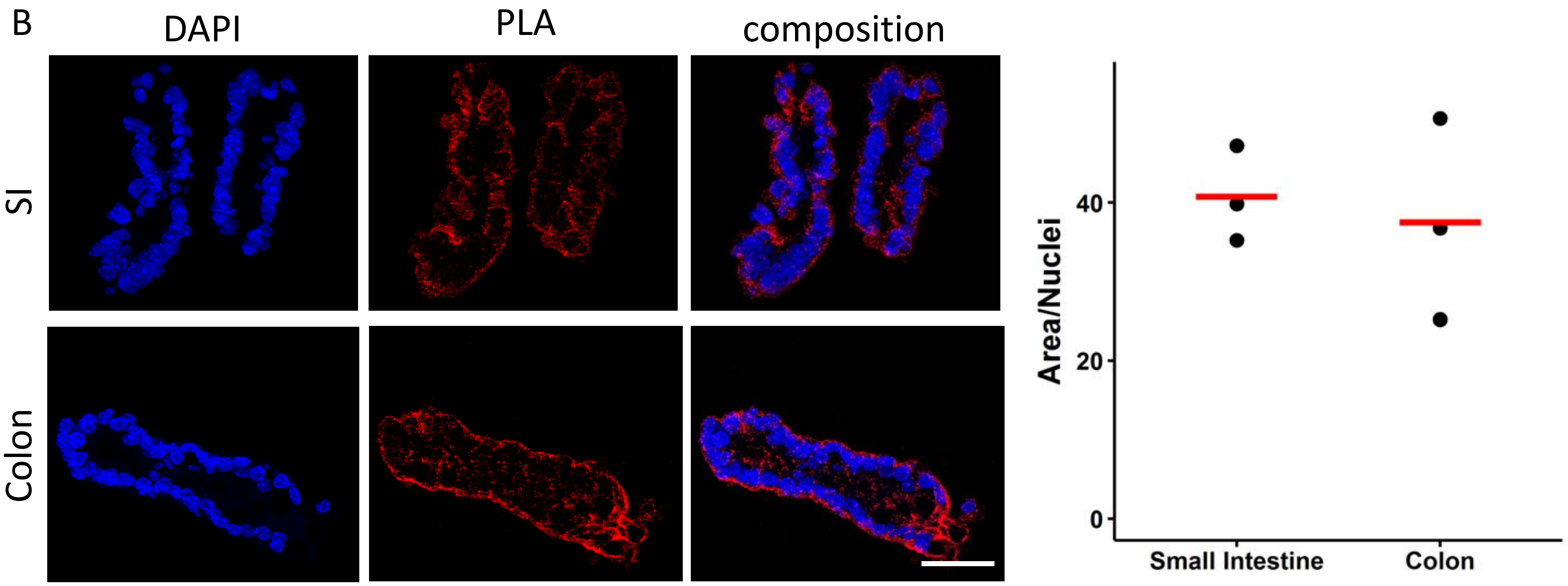

Appendix Fig. S6

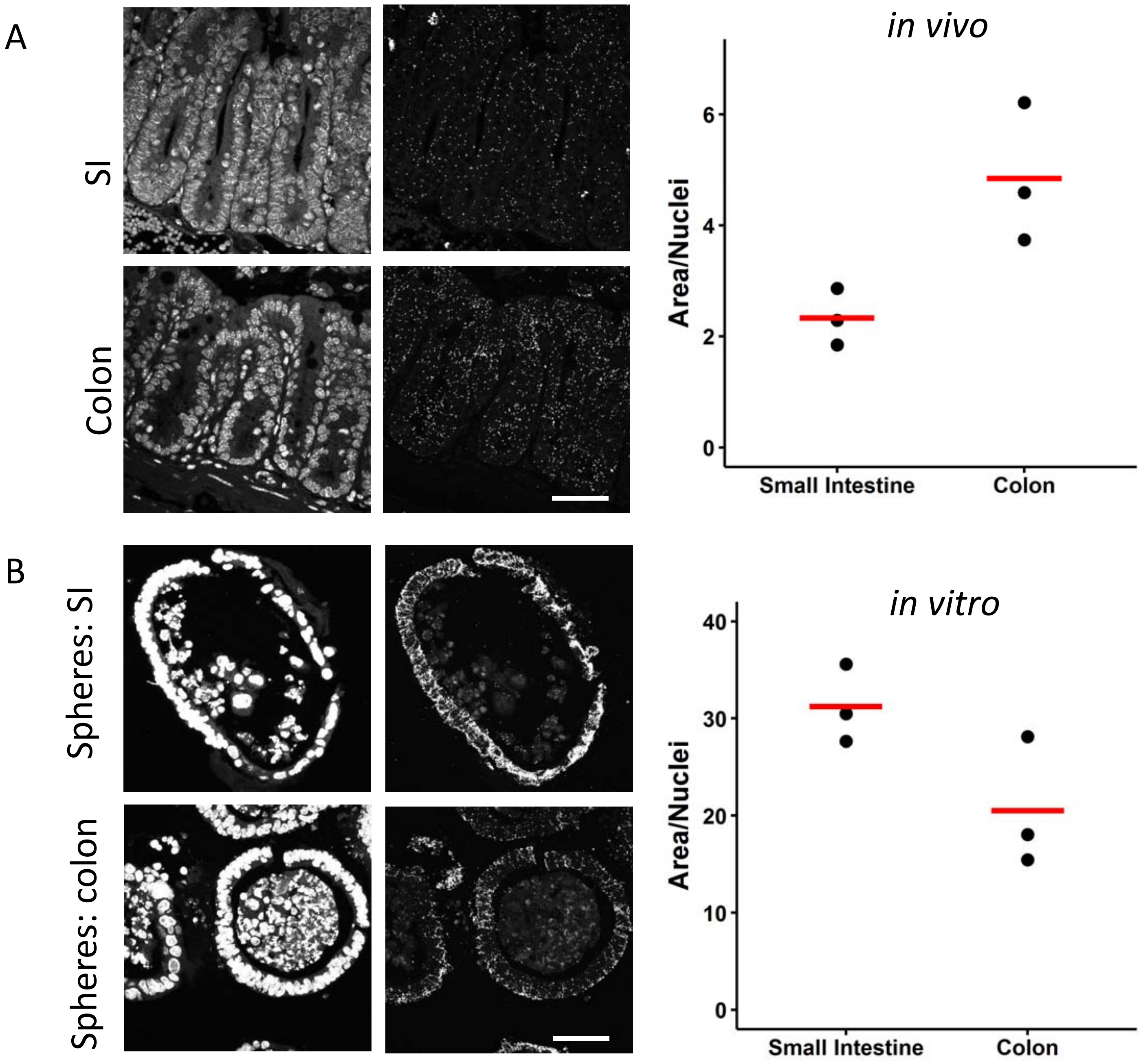

Supplement: Supplementary file 1 — Appendix [file embj0034-2321-sd1.pdf]
