# Supplementary material for: E-cadherin can limit the transforming properties of activating β-catenin mutations
Source: EMBO J. 2015 Aug 3;34(18):2321–33. doi: 10.15252/embj.201591739 (PMC4570519; doi:10.15252/embj.201591739)
Supplement: Supplementary file 2 — Expanded View Figures PDF [file embj0034-2321-sd2.pdf]

Expanded View Figures

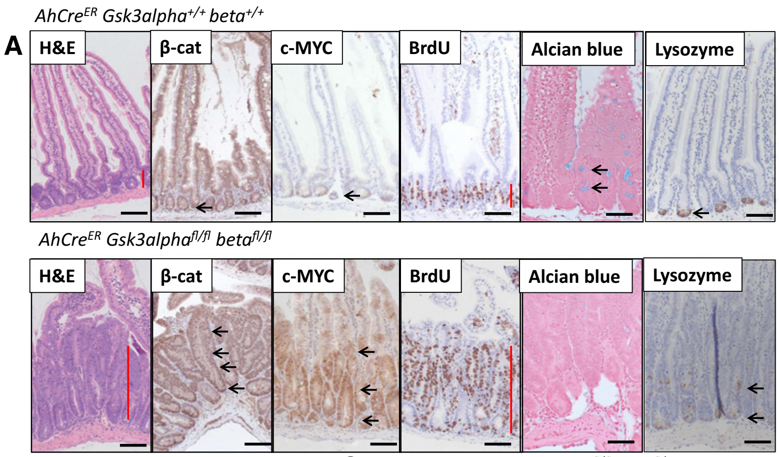

B

Cohort of ***AhCre Gsk3α Gskβ*** mice aged to a maximum of 600 days after induction.	
**Genotype**	**Mice with intestinal tumours**
Gsk3α $^{+/+}$ *Gsk3β* $^{fl/fl}$	0/8
Gsk3α $^{fl/+}$ *Gsk3β* $^{fl/+}$	0/10
Gsk3α $^{fl/+}$ *Gsk3β* $^{fl/fl}$	5/14
Gsk3α $^{fl/fl}$ *Gsk3β* $^{fl/+}$	3/6

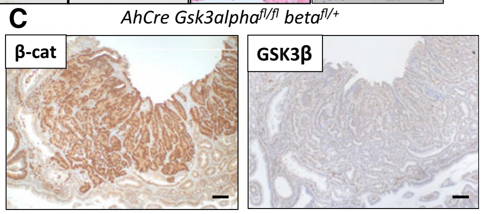

Figure EV1. Only deletion of both *Gsk3alpha* and *Gsk3beta* leads to crypt-progenitor-cell phenotype.

A Small Intestine of mice at day 6 after induction. Loss of Gsk3alpha and Gsk3beta (*AhCre*^{ER} *Gsk3alpha*$^{fl/fl}$ *Gsk3beta*$^{fl/fl}$) leads to accumulation of nuclear β-catenin and upregulation of cMyc (arrows). The crypt-progenitor cell phenotype (red bar) is characterised by increased proliferation (BrdU) and perturbed differentiation/localisation of goblet and Paneth cells (Alcian Blue, Lysozyme respectively, arrows). Scale bar, 100 μm.

B Table shows cohort of *AhCre Gsk3alpha Gsk3beta* mice aged until signs of intestinal tumour burden, genotypes as indicated. Note, mice homozygous for *Gsk3beta* deletion, or with only one copy of *GSK3alpha* and *GSK3beta*, did not develop intestinal tumours.

C An adenoma from an aged mouse deficient for 3 alleles of *Gsk3alpha* and *Gsk3beta (AhCre GSK3alpha*$^{fl/fl}$ *beta*$^{fl/+}$*)* showing that it developed after loss of the remaining GKS3beta allele. Scale bar, 100 μm.

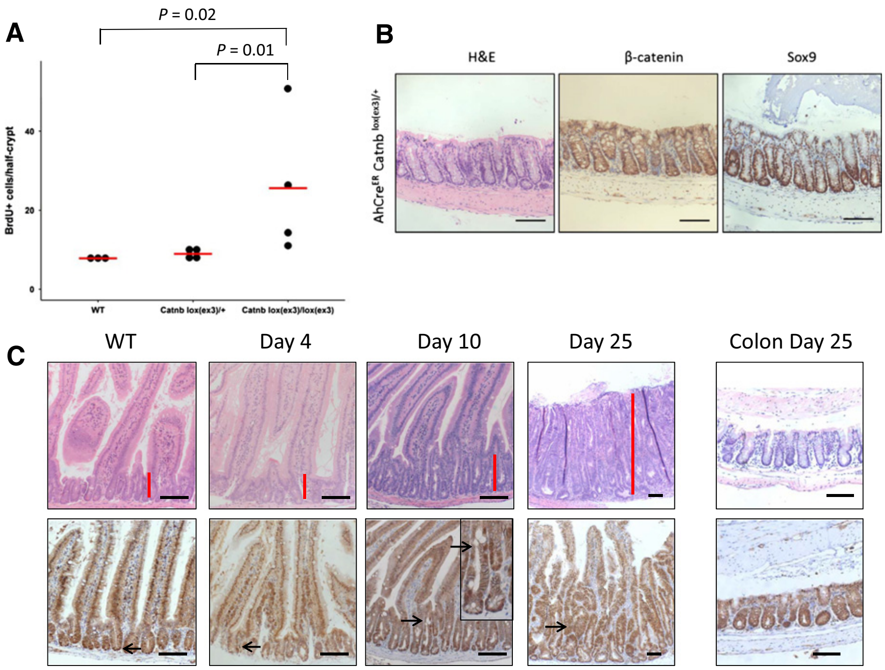

Figure EV2. Single copy activation of β-catenin only slowly transforms the intestine.

A Only activation of both alleles of β-catenin led to hyperproliferation in the small intestine. Proliferation of wild-type (WT), *AhCre*^{ER} *Catnb*$^{lox(ex3)/+}$ and *AhCre*^{ER} *Catnb*$^{lox(ex3)/lox(ex3)}$ mice 5 days after induction was scored by counting the number of BrdU-positive cells/half-crypt. $N \geq 3$ per group, at least 25 crypts per mouse were scored, *P*-value of one-sided Mann–Whitney *U*-test.

B Activation of one of copy β-catenin in an aged *AhCre*^{ER} *Catnb*$^{lox(ex3)/+}$ at day 25 post-induction with no phenotype in the colon. For comparison to a WT colon, see Appendix Fig S3. Scale bar, 100 μm.

C Activation of one copy of β-catenin in *VilCreER Catnb*$^{lox(ex3)/+}$ leads to the same crypt-progenitor cell phenotype (red bar) with similar kinetics as observed in *AhCre*^{ER} *Catnb*$^{lox(ex3)/+}$ mice. Scale bar, 100 μm.

 © 2015 Cancer Research UK Beatson Institute

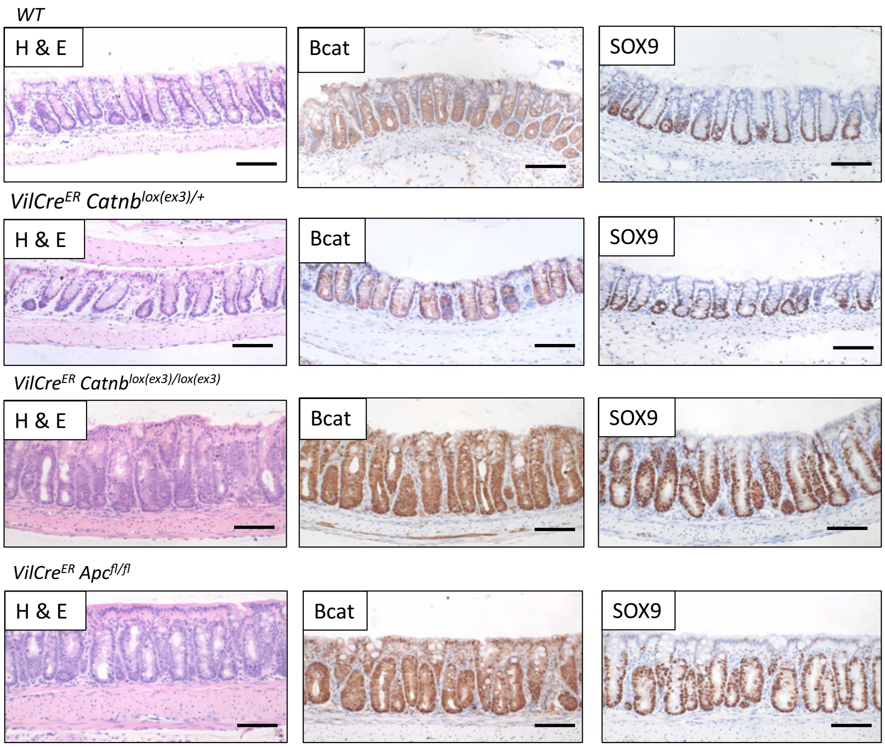

Figure EV3. Rapid colonic phenotype in *VilCre^{ER} Catnb$^{lox(ex3)/lox(ex3)}$* and *Apc$^{fl/fl}$* mice, but not in *VilCreER Catnb$^{lox(ex3)/+}$* mice.
Wild-type mice show very little nuclear β-catenin, and the expression of Sox9 is restricted to the bottom of the crypt. *VilCreER Catnb$^{lox(ex3)/+}$* show a similar phenotype. In contrast, *VilCre^{ER} Catnb$^{lox(ex3)/lox(ex3)}$* and *VilCre^{ER} Apc$^{fl/fl}$* mice show increased nuclear β-catenin and high expression of Sox9. Scale bar, 100 μm. Mice were sampled at day 4 after induction.

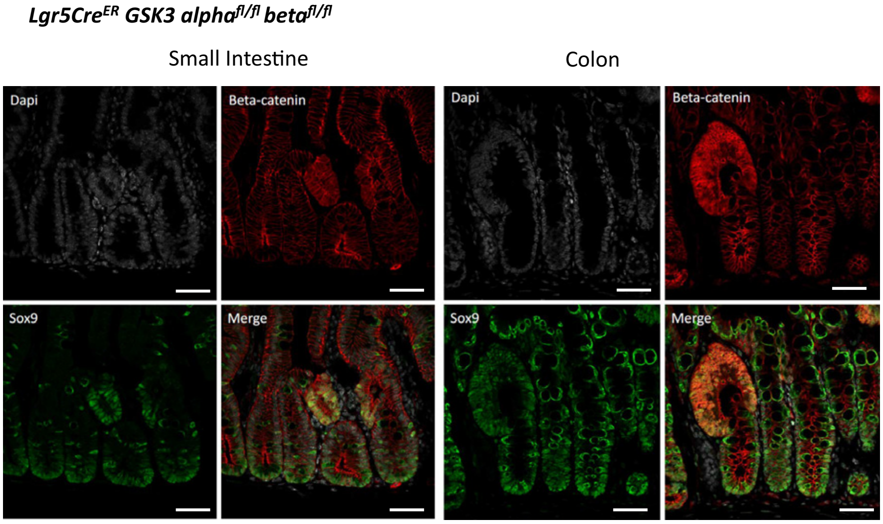

Figure EV4. Complete loss of Gsk3 in Lgr5-positive stem cells leads to tumour formation in the small intestine and the colon.
Immunofluorescence analysis shows intestinal lesions with accumulation of β-catenin and SOX9 in the small intestine and the colon, 25 days after induction. Scale bar, 50 μm.

© 2015 Cancer Research UK Beatson Institute

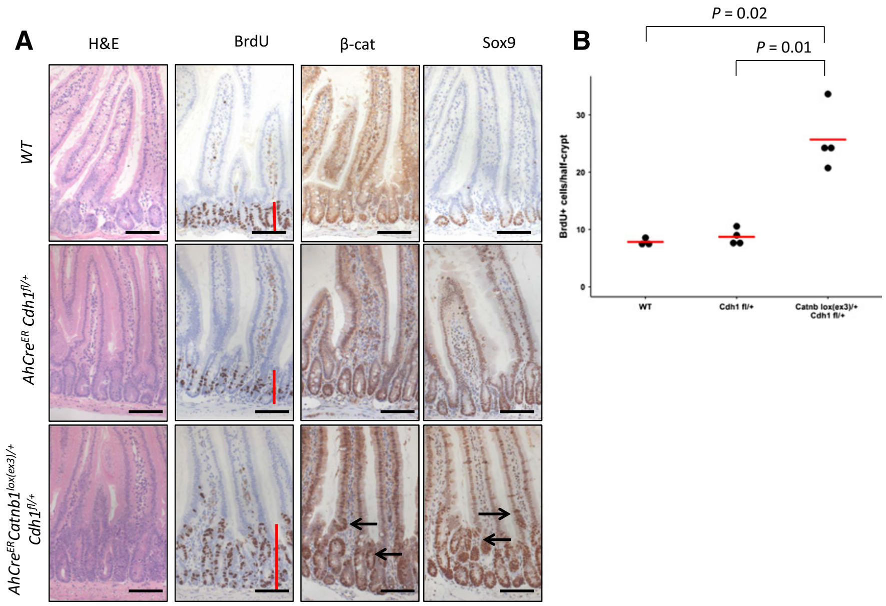

Figure EV5. Haploinsufficiency for E-cadherin lowers the threshold for Wnt activation of β-catenin mutation.

A *AhCre*^{ER} *Cdh1*$^{fl/+}$ at day 5 post-induction shows no intestinal phenotype. *AhCre*^{ER} *Catnb*$^{lox(ex3)/+}$ *Cdh1*$^{fl/+}$ mice show increased proliferation (BrdU, red line) and accumulation of β-catenin and Sox9 in cells higher up the crypt-villus axis (arrows). Scale bar, 100 μm.

B Increased proliferation as scored by BrdU^{+} cells per half-crypt. $N \geq 3$, statistics: one-sided Mann–Whitney *U*-test.

 © 2015 Cancer Research UK Beatson Institute
